# OsiriXGPT: An Innovative AI Co-pilot Plug-In for Seamless Deployment of Generative AI Models in Scan-to-Scan Reporting Workflows

**DOI:** 10.1007/s10278-025-01712-2

**Published:** 2025-10-16

**Authors:** Antonio Candito, Timothy Sum-Hon Mun, Richard Holbrey, Simon Doran, Christina Messiou, Dow-Mu Koh, Matthew D. Blackledge

**Affiliations:** 1 Division of Radiotherapy and Imaging, The Institute of Cancer Research, London, UK; 2 MRI Unit, Royal Marsden NHS Foundation Trust, London, UK

**Keywords:** OsiriX, OsiriXgrpc, Large Language Models (LLMs), Vision-Language Models (VLMs), One-click AI-driven segmentation tools, Radiology workflow, Radiology reporting

## Abstract

Generative Artificial Intelligence (GenAI) has the potential to transform radiology by reducing reporting burdens, enhancing diagnostic workflows and facilitating communication of complex radiological information. However, research and adoption remain limited due to the lack of seamless integration with medical imaging viewers. This study introduces OsiriXgrpc, an open-source API plug-in that bridges this gap, enabling real-time communication between OsiriX, a CE-marked and FDA-approved DICOM viewer, and AI-driven tools deployed in any supported programming language (e.g., Python). OsiriXgrpc’s design provides users with a unified platform to query, interact with, and visualise AI-generated outputs directly within OsiriX. To demonstrate its potential, we developed an AI Co-pilot for radiology that leverages OsiriXgrpc for iterative “request-to-answer” interactions between users and GenAI models, allowing real-time data queries and AI-generated output visualisation within the same DICOM viewer. We have adapted OsiriXgrpc to allow users to: (i) interrogate Foundation Large-Language Models (LLMs) to generate text from text-based prompts, (ii) employ Foundation Vision-Language Models (VLMs) to generate text by combining text and image prompts, and (iii) employ a one-click Foundation AI-driven segmentation model to generate Regions of Interest (ROIs) by combining points/bounding boxes with text prompts. For this proof-of-concept report, we applied OpenAI’s LLMs and VLMs for text generation and the Segment Anything Model (SAM) for generating ROIs. We provide evidence for successful implementation of the plug-in, including visualisation of the AI-generated outputs for each model tested. We hypothesise that OsiriXgrpc can lower adoption barriers, facilitating GenAI models integration into clinical trials and routine healthcare, even in resource-limited settings, including low/middle income countries (LMICs).

**Supplementary Information:**

The online version contains supplementary material available at https://doi.org/10.1007/s10278-025-01712-2.

## Introduction

The development of Generative Artificial Intelligence (GenAI) presents a unique opportunity to address unmet clinical needs in radiology. The growing number of patients, shortage of radiologists, increased scanner capacity, advances in cancer treatments, and the rising volume of medical imaging have led to increased workloads and mounting pressure on radiologists and radiology departments [1, 2]. GenAI models are demonstrating significant ability to analyse and interpret multi-modal medical data, offering radiologists an opportunity to embrace this technology to improve workflows and alleviate some of these burdens [3, 4]. This technology has the potential to improve the backlog in national healthcare systems, such as the National Healthcare Service (NHS), where a recent snapshot revealed that only 69.2% of cancer patients were treated within the 62-day target following an urgent referral in July 2025, significantly below the 85% target (available from https://www.england.nhs.uk/statistics/statistical-work-areas/cancer-waiting-times/). The bottleneck caused by delays and access to imaging diagnostics is a recognised contributing factor. However, a significant gap remains in deploying AI-assisted solutions, particularly in radiology, where the complexity of data and clinical workflow demands a higher level of integration and interoperability [5].

Despite the potential and transformative promise of GenAI models developed by high-tech companies such as OpenAI [6], Google [7], and Meta [8], translation of these technologies into certified medical imaging viewers is hindered due to regulatory and technological constraints. Instead, users may be tempted to upload data to web-based interfaces to generate AI-driven responses, significantly increasing the risk of security/General Data Protection Regulation GDPR breaches and limiting validation of these technologies on real-world clinical datasets [9, 10].

Although some companies have deployed task-specific AI models directly into hospital systems, these solutions are often purchased individually. This leads to inefficiencies in the procurement process, because of the requirement to engage in multiple, parallel, negotiations with a variety of AI suppliers to create data-sharing agreements and address well founded privacy and security concerns. Each legal agreement takes significant time and resource, leading overall, to unwanted delays in adoption. Further disadvantages of having multiple suppliers are: (i) an elevated cybersecurity risk because of the increased “attack surface” arising from potential compromises of the supply chain (e.g., the 2024 Synnovis cyberattack that affected several leading UK hospitals); and (ii) an increased burden on IT and Picture Archiving and Communication System (PACS) teams to integrate each AI-driven model with PACS for retrieving DICOM studies.

To address this issue, radiology departments are increasingly embracing innovative imaging AI *platforms* that host and facilitate using CE-marked and FDA-approved task-specific AI-driven models developed by third parties [11–13]. These platforms aim to act like “app stores” for radiology, enabling radiologists to choose from a variety of algorithms tailored to specific tasks, such as disease detection and classification, segmentation, and report generation, within their workflows [14–16]. By signing a single master contract with the platform provider, procurement becomes more efficient, and accountability for cybersecurity is simplified.

Such platforms currently integrate with PACS, primarily via the “AI reports” generated by the individual “apps” and stored back into PACS by the platform in the form of secondary capture DICOM or PDF “wrapped” into DICOM. However, a weakness of this model is that, currently, imaging AI platforms are not integrated into the PACS workstations themselves. As a result, radiologists often rely on a second screen to view and assess retrieved DICOM studies, and they interact with AI-driven models via proprietary software created by each individual app vendor. Additionally, the image viewer, interfaces and toolboxes provided by the platforms differ from those in PACS. These fragmentations and differences can disrupt radiologists’ workflows, potentially neutralising the efficiencies promised by the AI-driven models or even increasing reporting time.

Currently, there are no open-source solutions that enable the dynamic deployment and implementation of GenAI models within a certified medical imaging viewer. This presents a significant challenge for developers and radiologists who need a unified platform to collaborate, test, validate, and refine GenAI models for regulatory approval. Without such a solution, developers lack an integrated environment where users can provide valuable feedback on model performance in scan-to-scan reporting workflows, which would helping to collect evidence, reduce bias, and enhance trustworthiness in future iterations. A standardised, open platform could lower costs and complexity in generating the necessary evidence and documentation for FDA approval of innovative multi-modal biomedical AI solutions.

In this paper, we firstly introduce an open-source project, OsiriXgrpc, an API plug-in (available via [*blinded for review]*), which facilitates rapid development of medical image analysis tools into the popular radiological viewing platform, OsiriX [17], using any supported language (including Python). Integrating the latest technology in remote procedure call (Google Remote Procedure Call, gRPC) allows us to leverage the extensive experience of the OsiriX team in creating a widely disseminated, low-cost, user-friendly interface in order to deliver a ready-made environment within which to install cutting-edge workstation AI apps.

As an exemplar use-case, demonstrating the advantages of OsiriXgrpc as a complementary paradigm to existing AI platform technologies, we subsequently propose a novel AI Co-pilot application-level plug-in for assisting radiologists in reporting findings from medical imaging and providing quantitative analyses on-demand, and in a standardised manner. Our “all-in-one” plug-in facilitates iterative interactions between radiologists and GenAI models by enabling users to make requests and receive AI-generated outputs, including conversational AI responses, screenshots of visualised medical images, AI-driven segmentations of clinical findings, and associated radiological measurements. GenAI models can assist in interpreting multi-modal medical data, and the plug-in allows users to interact with Foundational Large-Language Models (LLMs), Vision-Language Models (VLMs), and Foundation AI-driven segmentation tools either using text prompts or speech input, or by combining such queries with images and regions-of-interest (ROI) [18–21]. Furthermore, users can visualise and manipulate images, as well as view AI-generated outputs, directly on a CE- and FDA-certified DICOM viewer, eliminating the need for a dedicated AI-vendor workstation or learning specific interfaces for individual AI models/software tailored to specific tasks. In addition, we fine-tuned a foundation segmentation model for use with whole-body diffusion-weighted MRI (WB-DWI) of patients with metastatic advanced prostate cancer (APC) and multiple myeloma, and deployed it as an additional toolbox within OsiriX for testing by an independent radiologist.

It is important to emphasize that the present work focuses on software integration and workflow optimization, rather than the development of new AI algorithms. OsiriX can be configured to communicate with PACS, allowing radiologists to retrieve medical studies directly within OsiriX and seamlessly query and interact with GenAI models via the AI Co-pilot interface. This approach parallels ongoing efforts in PACS and health IT integration [22, 23]. From a human–computer interaction (HCI) perspective, our interface design builds upon prior studies of radiology user interfaces and multimodal interaction [24]. Moreover, the OsiriXgrpc API plug-in aligns with emerging work on workflow interoperability and open-source radiology systems [25, 26].

Our aim is to present how this integration can eventually enable radiologists to evaluate newly developed GenAI models in research projects and clinical trials for scan-to-scan reporting, as well as explore their potential role in routine clinical workflows.

## Materials and Methods

### Patient Dataset

To evaluate the implementation of the AI Co-pilot deployed within OsiriX via the OsiriXgrpc plug-in, we used a dataset consisting of one volunteer (screening study) and 14 datasets from patients diagnosed with Advanced Prostate Cancer (APC) who underwent whole-body MRI (WB-MRI) scans. We selected nine non-consecutive axial images from the 3D stack per study, excluding images covering patient faces. All data, including those employed for segmentation model fine-tuning (described in **Supplementary Section S1**), were fully anonymised, and the study was performed following the Declaration of Helsinki (2013). A local ethical committee waived the requirement of patient consent for the use of these retrospective datasets.

### OsiriXgrpc: An API Plug-in for Interoperability

The gRPC architecture is an open-source, high-performance remote procedure call (RPC) framework developed by Google that is compatible with any enabled environment (available from https://grpc.io/). It utilizes language-neutral protocol buffers to serialize messages, enabling seamless server-client communication across various systems and programming languages. gRPC has quickly become a leader in RPC technologies owing to its high performance and low latency, achieved using the HTTP/2 standard and efficient binary serialization of messages, as well as its ease of integration across multiple programming languages. Given that parsing of medical images is intrinsically a high-data-volume problem, we chose to implement gRPC as the RPC framework of choice in the design of OsiriXgrpc.

OsiriXgrpc represents a significant update to a previous solution, pyOsiriX [27]. By leveraging gRPC, it facilitates efficient inter-process communication between OsiriX and other systems. The OsiriXgrpc API retrieves pixel data and ROI objects from the active OsiriX viewer, serializes them into protocol-buffer messages, and streams them to the Python client, on average within 6 ms [28]. On the client side, these objects are typically converted to NumPy arrays for preprocessing (e.g., DICOM to JPEG conversion, ROI overlay), then passed through the “gatekeeper” (“Data handling and privacy safeguards” section) before either cloud-based or self-hosted inference. The API handles 2D single-frame images and multi-frame stacks. ROI types include polygons, ellipses/rectangles, freehand shapes, and text annotations. For 3D series, per-slice masks are returned with consistent indices, spacing, and orientation, enabling volumetric reconstruction on the client side.

The gRPC service can be hosted on the same hardware as the OsiriX viewer itself, on a GPU-enabled computer or cluster (possibly running a different operating system), or even a cloud-based platform, all connected via a network. One of the critical advantages of this technology is its flexibility: it allows users to work with any Python version and integrate relevant third-party research libraries such as TensorFlow [29] and PyTorch [30]. This integration promotes productive collaboration with expert clinicians and provides valuable feedback on model accuracy, facilitating iterative model refinement. This project is open-source and freely available to the imaging research community, fostering advancements in medical imaging and AI-driven healthcare solutions.

### OsiriXGPT: An AI Co-pilot Interface for Multimodal Radiology Workflows

**Fig. 1:**
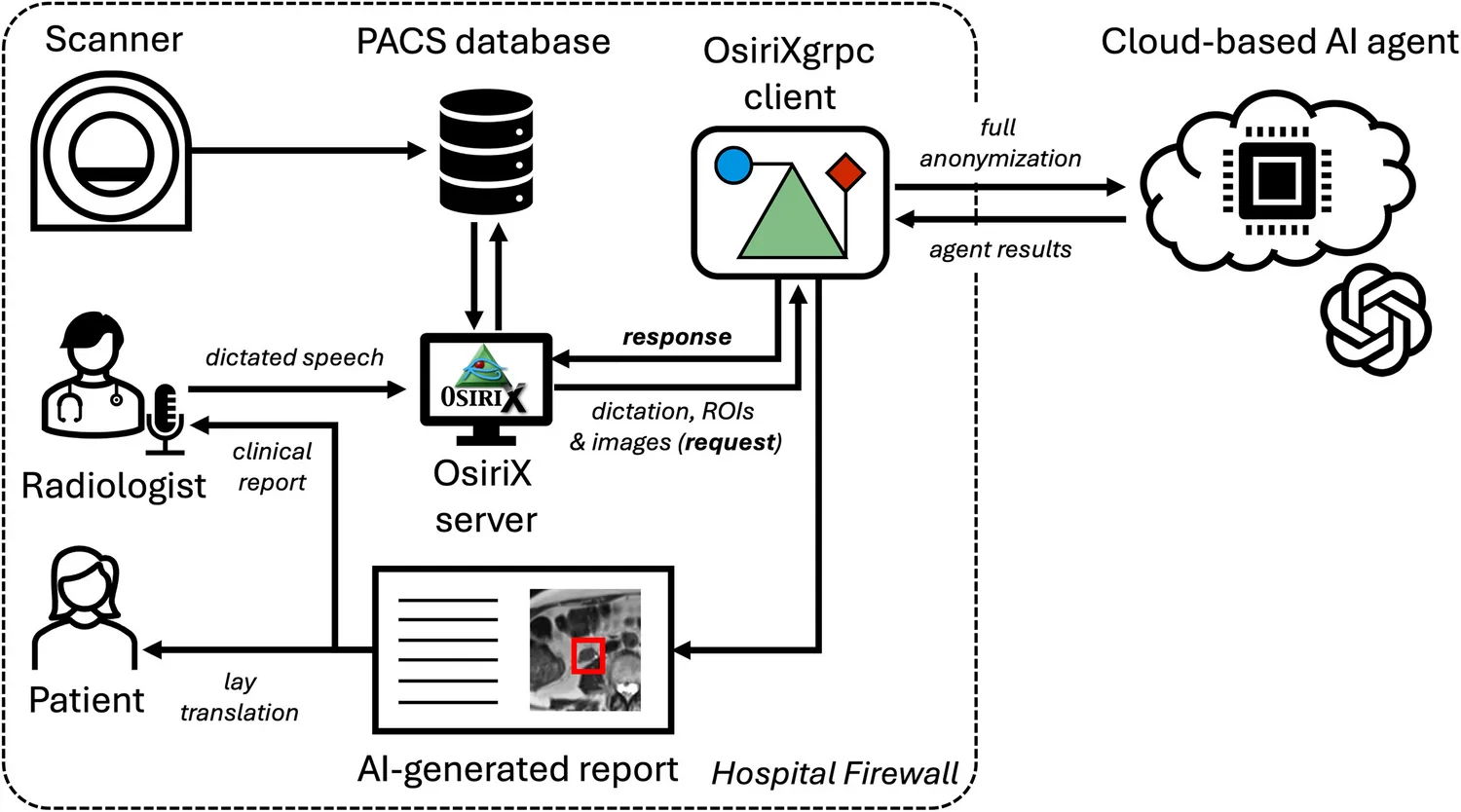
Overview of the AI Co-pilot interface integrated within a DICOM viewer workstation, enabling seamless iterative “request-to-answer” interactions with GenAI models to assist radiologists in interpreting multi-modal medical data and performing multiple tasks. The primary aim is accelerating and enhancing text-based report generation without disrupting the conventional scan-to-scan reporting workflow. Users can input instructions for GenAI models via keyboard input or dictation. Queries may include text prompts, visual prompts, or patient-centric data (e.g., laboratory test results). Supported visual prompts include 2D and 3D images, multiple imaging sequences, and longitudinal scans, even with associated ROIs (e.g., points and bounding boxes). These prompts can be queried, manipulated, and visualised directly within the OsiriX DICOM viewer, a CE- and FDA-approved tool widely used by clinicians. The OsiriXgrpc API plug-in facilitates communication between the imaging viewer and a Python environment responsible for GenAI model inference, locally on a GPU or via cloud-based instances (e.g., OpenAI-hosted language and vision models). GenAI models support text generation from text prompts, extracting insights from image-based prompts, and performing organ or disease delineation using text/ROI inputs

To demonstrate a use-case of OsiriXgrpc, we present OsiriXGPT, an open-source interface that acts as a bridge between an OsiriX workstation and GenAI models running on either a local GPU or a cloud server (see Fig. 1, GitHub repository will be publicly accessible in the near future). This tool aims to facilitate research on the use of GenAI in radiology following the principles of FAIR research software (Findable, Accessible, Interoperable and Reusable) [31].

Radiologists can leverage GenAI models to enhance various aspects of their workflow. For instance, AI-based solutions can assist in generating text to reduce reporting time [32], generating summaries and plain-language reports to improve communication of clinical findings with patients [33], and integrating images with clinical or laboratory test results to assist in resolving complex diagnoses [34]. Therefore, our plug-in is designed to interact with GenAI models developed for solving multiple tasks while also facilitating the handling of different input/output formats, as illustrated in Fig. 1. Radiologists may: (i) interrogate LLMs to generate text from text-only prompts; (ii) employ VLMs to generate images from text-based inputs; (iii) combine text prompts with images to generate ROIs using AI-driven segmentation tools; and (iv) leverage VLMs to generate description of voxel characteristics by a combination of text, images, and ROIs input prompts. All associated imaging data are intrinsically stripped of any patient-identifiable information as only voxel values (NumPy arrays) are sent to the OsiriXgrpc-connected Python environment, where scripts are executed for image processing or GenAI models inference. All processed outputs are returned to the OsiriX workstation for display.

Furthermore, an important feature of OsiriXGPT is that compared with conventional workflows, radiologists can iteratively interact with the GenAI models. Using text and/or images prompts to generate an initial output, they can then evaluate the response based on its accuracy and relevance providing where necessary additional guidance, clarifying instructions, providing more data, or asking follow-up questions. GenAI models then reprocess the new input and refine the output. Once the output is finalised, radiologists can include the AI-generated text with the radiological report which could, for example, then be saved in the Radiology Information System (RIS) where required.

In this study, we integrated OpenAI’s GenAI models (LLMs, VLMs, and speech recognition algorithms) [35, 36] into our workflow. Since these models are not available for local inference, input prompts (such as text, images, or audio) are sent to OpenAI’s cloud servers for processing. This entire process, submitting prompts and receiving AI-generated outputs, is managed through the OpenAI API, within the Python environment, and facilitated by OsiriXgrpc.

The OpenAI API enables a structured request-response dialogue between “User” and “AI Assistant” (represented by the GenAI models). Before processing any prompt, the AI Assistant is assigned a contextual role: “*Your name is OsiriXGPT: an AI Co-pilot for radiology. You are a highly skilled assistant for a radiologist, specialised in analysing medical images and generating comprehensive, detailed reports. Do not leave any prompt unanswered, especially when asked to report findings from medical images*”. To ensure coherent and contextually relevant responses, a conversation history from the same chat (including all previous User and AI Assistant dialogues) is combined with the current text prompt for each query.

We used the multimodal OpenAI model gpt-4o (snapshot: gpt-4o-2024–11–20) accessed via the REST API (v1) through the OpenAI Python SDK (v0.28.0). We set temperature = 0.1 and max tokens = 600, while leaving all other decoding parameters at their default values (available from https://platform.openai.com/docs/api-reference/). A lower temperature value makes responses more deterministic, while a higher value increases randomness and variability in outputs. The maximum token limit determines the length of the response, with higher limits allowing for more detailed answers but also increasing cost, as pricing is typically based on the number of tokens used. For querying VLMs, DICOM images are converted to JPEG files, which are then encoded as Base64 strings and paired with corresponding text prompts for inference.

Additionally, we integrated META’s Segment Anything Model (SAM) [37], an AI-powered segmentation tool, into the workflow. To generate a segmentation, users can simply draw points or a bounding box within or around the region of interest using OsiriX’s built-in toolbox. DICOM images and associated prompts undergo a pre-processing pipeline before generating a prediction. The pre-processing steps involve converting the DICOM file to a NumPy array, normalizing the image, converting it to an 8-bit RGB format, and resizing it to 1024 × 1024 pixels. To ensure spatial consistency, user prompts (points, bounding boxes, or masks) are resampled to the same resolution as the input image. Both the image and prompt are then passed to the model’s encoder to generate feature embeddings. The model processes these embeddings to generate an output mask, which is displayed in the OsiriX viewer and for extracting quantitative measurements within delineated regions. OsiriXgrpc once again facilitates the data exchange between OsiriX and the Python environment, where the segmentation model is installed, and image processing scripts are implemented. Since SAM’s weights and biases are available for download, the inference process is performed locally.

To implement the AI-based speech recognition tool, we ensured that the audio recording is performed at a sampling rate of 44.1 kHz with a 10-s reading time window from a preferred audio input device. The recorded audio data is temporarily stored in Waveform Audio File (WAV) format and encoded in 16-bit Pulse Code Modulation (PCM) before being processed by OpenAI’s Whisper-1 model for speech-to-text conversion. The system continuously listens and transcribes in real time, concatenating the output text until the keyword “STOP” is detected, automatically terminating the recording process.

All algorithms were implemented in Python (v3.9.19) using OsiriX (v0.2.1-beta5), pyOsiriX (v0.2.1-beta5), OpenAI (v0.28.0), pydicom (v2.4.4), SAM (v1.0.0), sounddevice (v0.4.7), PyTorch (v2.3.0), SimpleITK (v2.2.1), and NumPy (v2.0.0) libraries, running on a MacBook Pro (including the local inference of the semi-automated segmentation models). To minimize drift across software or model updates we:

1. Pinned a dated model snapshot (gpt-4o-2024–11–20),
2. Froze the Python environment by exporting a full Conda specification (environment.yml), capturing the Python version and all package dependencies,
3. Fixed decoding settings (e.g., temperature) and logged all request details, including the model ID, parameter values, full prompt text, image preprocessing steps, and token counts, and
4. Implemented evaluation tests using prompts and images benchmarked against ground-truth outputs to detect shifts in model responses after software or model updates, following OpenAI’s recommended evaluation practices (available from https://platform.openai.com/docs/guides/evals?api-mode).

**Fig. 2:**
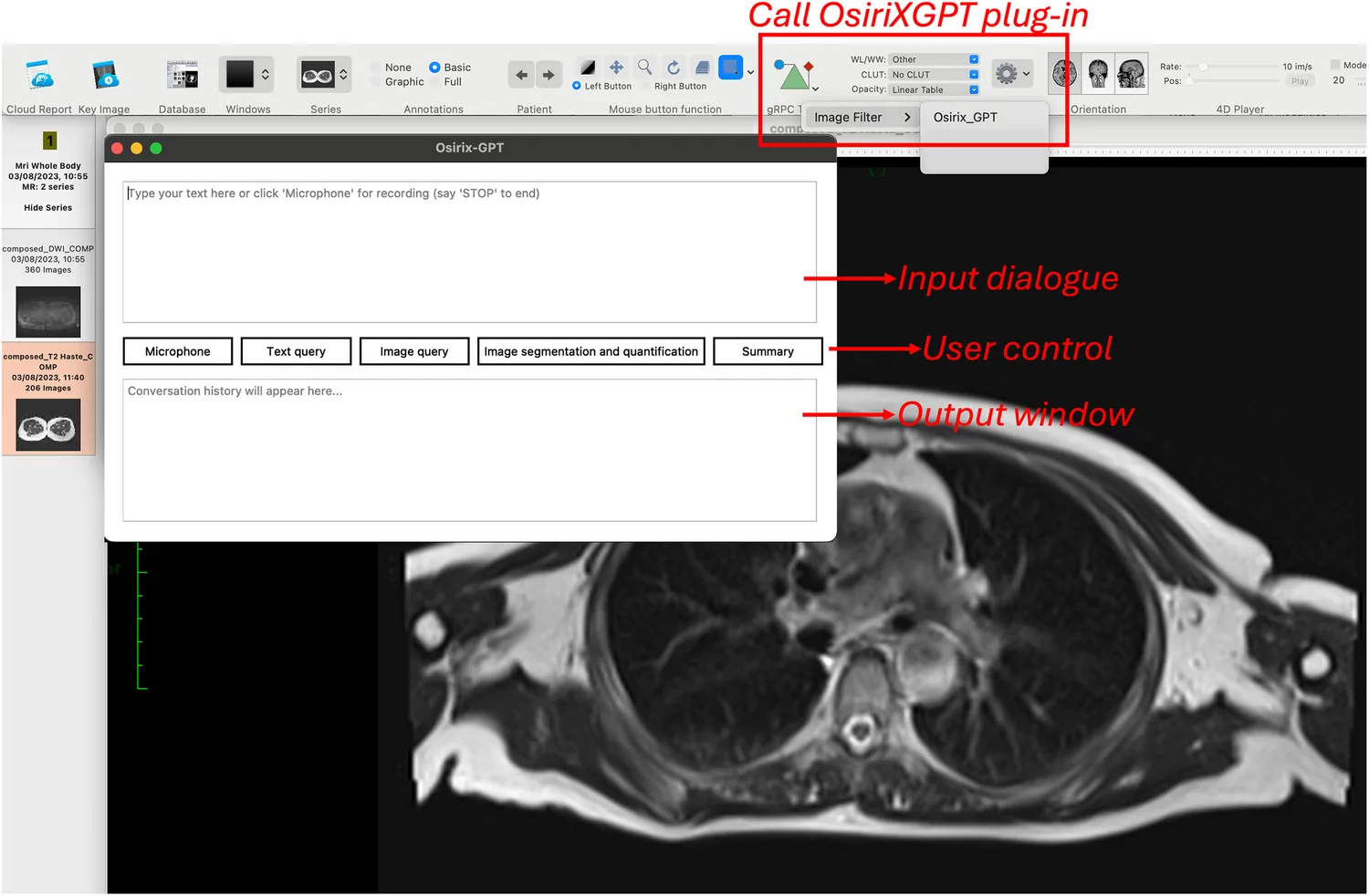
Screenshot of the 2D DICOM viewer in OsiriX showing the AI Co-pilot interface, OsiriXGPT, available as an additional toolbox. Upon activation, the toolbox launches a dedicated GUI (“OsiriXGPT”) where users can interact with GenAI models by submitting requests via an *input dialogue*. Requests are executed using five control buttons in the GUI’s *user control* panel, which enable GenAI models to interpret and process text prompts (e.g., generating text-based outputs via the “Text query” control) through the OsiriXgrpc API plug-in. Text prompts can also be associated with input images and ROIs displayed in the 2D imaging viewer for tasks such as image analysis or segmentation mask generation (via the “Image query” and “Image segmentation and quantification” controls). The AI-generated output is displayed in the GUI’s *output window*. OsiriXGPT also allows users to export requests and corresponding AI outputs as a PDF file using the “Summary” control. Additionally, the “Microphone” control enables speech-to-text input, eliminating the need for manual typing

An overview of the simple and flexible graphical user-interface (GUI) for OsiriXGPT is presented in Fig. 2. The GUI is divided into three sections: (i) an ***input dialogue*** that allows users to input text prompts via the keyboard, (ii) the ***user control*** including five buttons for interacting with various GenAI models using the prompts, and (iii) ***output window*** that displays the AI-generated responses (a record of user inputs is differentiated from AI-generated responses via the “User” and “Assistant” keywords respectively).

Each of the five buttons in the *user control* serves a specific function:

- **Microphone**: Allows users to dictate text prompts instead of typing. It uses an AI-based speech recognition tool to convert speech into text, which is then displayed in the *input dialogue*.
- **Text query**: Sends the text input to the LLMs to generate a text-based response.
- **Image query**: Enables interaction with VLMs by sending both text prompts and associated images, which are visualised and manipulated by users using the 2D OsiriX viewer toolbox.
- **Image segmentation and quantification**: Triggers the segment anything model (SAM) to generate segmentation masks based on text prompts and manually defined points/bounding boxes within/around ROIs. The resulting mask is displayed in the OsiriX viewer, and corresponding measurement statistics (mean and standard deviation) from the semi-automated delineations are shown in the *output window*.
- **Summary**: Exports the conversation between the User and Assistant as a PDF report. The report includes the displayed images from the 2D OsiriX viewer toolbox, along with any superimposed masks generated by the user or AI-driven segmentation tools.

### Data Handling and Privacy Safeguards

Using the API of OsiriXgrpc, we have rapidly implemented a dedicated “gatekeeper” process that runs locally on the same workstation as the original data and mediates all communication with the OpenAI API. Its role was to ensure that only de-identified JPEGs generated by the DICOM-to-JPEG conversion pipeline were submitted to OpenAI, thereby preventing raw DICOM files or other sensitive data from leaving the local environment. During conversion, visual contrast and user-defined ROIs were preserved, while all metadata fields (e.g., patient name, ID, date of birth) that may contain Protected Health Information (PHI) were stripped out.

Because PHI can also appear within the pixel data itself (e.g., scanner overlays or burned-in text labels), the gatekeeper was extended with an automated Optical Character Recognition (OCR) redaction step. The Presidio Image Redactor Python library (v0.0.57) was implemented to detect and mask text regions within DICOM images [38]. To evaluate this approach, we generated synthetic patient information (names and dates of birth) and burned it into 5 randomly selected slices from whole-body b900, ADC, and T2 HASTE DICOM images of 14 patients scanned with multi-parametric whole-body MRI (Sect. 2.1), resulting in 210 tests in total.

### Initial Evaluation

To test the workflow enabled by OsiriXGPT we used WB-MRI case study acquired for detecting cancer across the body in an asymptomatic high-risk patient. WB-MRI is an advanced, radiation-free, and non-invasive diagnostic technique that allows the quantification of imaging biomarkers for assessing both benign and malignant lesions [39–41]. However, the large number of images and MRI sequences acquired increases reporting time and requires highly trained radiologists. Reporting clinical findings from a WB-MRI study can be more challenging compared to conventional imaging focused on specific organs. Therefore, our OsiriXGPT plug-in may be valuable in assisting radiologists with reporting WB-MRI for cancer detection by potentially reducing reporting time, resolving uncertainties in clinical findings, and generating structured reports.

We benchmarked OsiriXGPT through three initial tests to verify correct implementation in processing text prompts and validate the speech-to-text capability, querying data from OsiriX 2D viewer, and generating outputs using OpenAI’s GenAI models and SAM. The subsequent two tests demonstrated how OsiriXGPT can assist radiologists in delivering structured reports for WB-MRI volunteer studies.

- **Test 1:** To demonstrate connectivity with OpenAI through the OsiriXGPT interface and showcase the potential of OsiriXGPT for radiologist support, we generated a response from the LLMs using the text prompt: “*Provide a summary how to use the ONCO-RADS score system guidelines for reporting WB-MRI for cancer screening*”. This highlights the capability of integrating AI-driven tools to streamline guideline interpretation *within the same radiological viewing platform*, enabling radiologists to save time, improve consistency in reporting, and enhance patient care through more efficient workflows. We also tested the AI-based speech recognition tool by asking five volunteers from our research group to dictate the sentence: “*The quick brown fox jumps over the lazy dog*” (a pangram). We then calculated the Word Error Rate (WER), where lower values indicate higher accuracy in the speech-to-text conversion.
- **Test 2:** We generated a response from the VLMs by providing a text prompt, along with associated images and ROIs, asking, “*Which organ is visible within the delineated bounding boxes on this T2-weighted MRI?*”. For this test, we manually defined bounding boxes for the kidneys on clinical T2-weighted (T2w) Half-Fourier Acquisition Single-Shot Turbo Spin Echo (HASTE) MRI. Additionally, we assessed the accuracy of the VLMs in classifying anatomical structures such as the bladder, kidneys, liver, spleen, vertebral bodies (L3, T6, C3), heart, and brain using the text prompt, “*Which primary soft tissue organs or skeletal regions are visible within the delineated bounding box(es) on this T1-weighted HASTE MRI?*” For this evaluation, we used 14 WB-MRI datasets from patients diagnosed with APC. Bounding boxes for each structure were manually defined on the mid-slice on T2-weighted HASTE images prior to interrogating the VLMs via the OsiriXGPT plug-in. The plug-in was restarted for each patient to prevent the model from leveraging context or patterns from prior analyses, ensuring an unbiased response for each dataset.
- **Test 3:** To demonstrate the utility of our tool for semi-automatic segmentation using SAM, we attempted delineation of the spleen and liver on T2w HASTE images using the text prompt, “*Generate the segmentation of the spleen and liver*”. This requires delineation of bounding boxes around the requested regions, which are easily defined using the various OsiriX ROI tools.
- **Test 4:** We generated a response from the VLMs by providing a text prompt, along with the associated b900 diffusion-weighted (b = 900 s/mm^2^) and T2w HASTE images, and ROIs of a suspected lesion, asking to, “*Analyse the lesion within the red bounding boxes on the provided b900 DWI and T2w HASTE images. Describe: signal intensity, morphology, surrounding anatomy, ONCO-RADS score*”.
- **Test 5:** Finally, we ensured that the GUI could record the radiologist-AI dialogue using the PDF generation feature of the GUI and providing a text prompt asking, “*Provide a structured report that includes clinical findings with measurements from the assessed organs, details of suspected lesions, and the suggested ONCO-RADS score*”. To simulate a dialogue (request-and-response) between the user and the GenAI models through the OsiriXGPT plug-in, the following steps were performed prior to generating a summary of the findings through LLMs inference: (i) delineations of the spleen and liver were generated using the one-click AI-driven segmentation tool (SAM), and their corresponding measurements (e.g., area and MRI signal intensity within the delineated regions) were derived; (ii) text describing the clinical condition of these organs, identifying any abnormalities, and assigning ONCO-RADS scores was generated by interrogating the VLMs; (iii) the one-click AI-driven segmentation tool was used to generate the delineation and associated measurements of the lesion; and (iv) a text description, including the lesion’s morphology, MRI signal intensity, and ONCO-RADS score, was generated by interrogating the VLMs. We also generated a “layperson summary report” from the recorded request-to-answer dialogue between User and Assistant for testing the potential of using LLMs thought our OsiriXGPT interface to eventually improve communication of findings between clinicians and patients.

For all tests, we estimated the latency of OsiriXGPT for processing the input defined by the user and display back the AI generated response in the GUI’s *output window*.

### OsiriXgrpc Plug-In for Whole-Body DWI SAM Segmentation

To demonstrate the flexibility of OsiriXgrpc to incorporate additional AI models, even those developed in-house, we fine-tuned and implemented a purpose-built SAM for semi-automatic delineation of whole-body diffusion-weighted MRI (WB-DWI) in oncology (here referred to as WB-DWI SAM). Full details of the fine-tuning mechanism and evaluation of this model is provided in **Supplementary Material, Section S1**. For implementation within OsiriXgrpc we developed a toolbox that allows users to interact with this fine-tuned model by defining a bounding box using built-in OsiriX functionality and a button to subsequently run the algorithm. To evaluate the functionality of this tool an expert radiologist manually delineated a single-slice region of interest (ROI) around 5–7 spatially distinct lesions in whole-body DWI studies of 12 patients with confirmed APC (N = 6) or focal myeloma (N = 6). They subsequently delineated a bounding box around the lesion on the same slice and used the trained model to derive semi-automated disease segmentations. They did not modify these delineations once complete. Considering each lesion as independent and using conventional calculation methods [42], we derived Bland–Altman statistics, including the repeatability coefficient (RC), intra-class correlation coefficient (ICC), percentage limits of agreement (LoA), and percentage coefficient of variation (CoV), comparing the accuracy of median ADC measurements from semi-automated and manually defined ROIs. We also derived standard morphological segmentation metrics: dice-similarity coefficient (DSC), Hausdorff distance (HD) and segmentation sensitivity (the proportion of positively identified pixels from manual contouring correctly classified as disease by the WB-DWI SAM model). We compared metrics between myeloma and metastatic prostate cancer independently.

## Results

### Testing of Data Handling and Privacy Safeguards

The OCR module successfully identified and redacted these labels with an accuracy of 90% (189/210 slices), demonstrating the feasibility of this tool for reducing the risk of sharing pixel-embedded identifiers (example figures are shown in **Supplementary Figures, **Fig. S1).

### Initial Evaluation

**Fig. 3:**
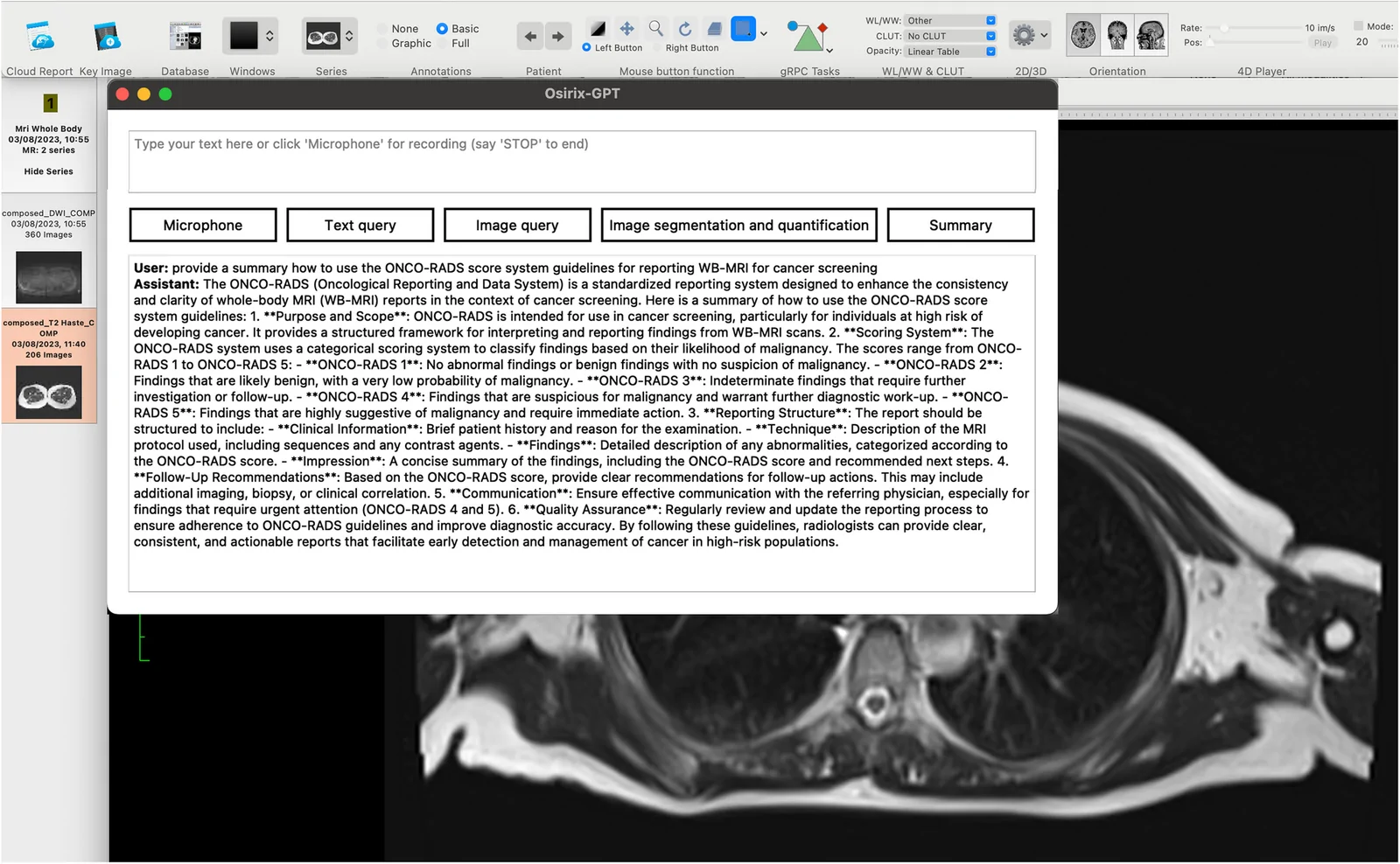
Screenshot of AI-generated text displayed in the *output window* of the OsiriXGPT GUI after querying LLMs with the text prompt: “*Provide a summary of how to use the ONCO-RADS score system guidelines for reporting WB-MRI for cancer screening*”. The prompt was entered into the GUI’s *input dialogue* and processed using the “Text query” control in the *user control* panel. OsiriXGPT leverages the OsiriXgrpc framework to facilitate communication between OsiriX and a Python environment where the OpenAI API is installed, enabling inference by LLMs on cloud-based servers. This process was performed while viewing medical images in the OsiriX 2D DICOM viewer. The OsiriXGPT interface successfully processed the query, and the LLMs provided a detailed description of the ONCO-RADS guidelines

**Test 1**: OsiriXGPT successfully processed the input and generated a summary of the ONCO-RADS guidelines [43], which appeared in the *output window* of the GUI within 7 s (see Fig. 3 for an example of the output). This confirmed the successful integration of cloud-based OpenAI API with the OsiriXGPT interface, ensuring fast and reliable response generation. Additionally, the speech-to-text “Microphone” feature in the GUI converted the user’s voice input into text, automatically displaying it in the *input dialogue* with a latency of less than 10 s. We also obtained an average WER of approximately 5% across data from our five volunteers. This allowed the user to view the dictated instructions in real time, confirming the successful integration of the OpenAI API for Audio-Speech model inference using our plug-in.

**Fig. 4:**
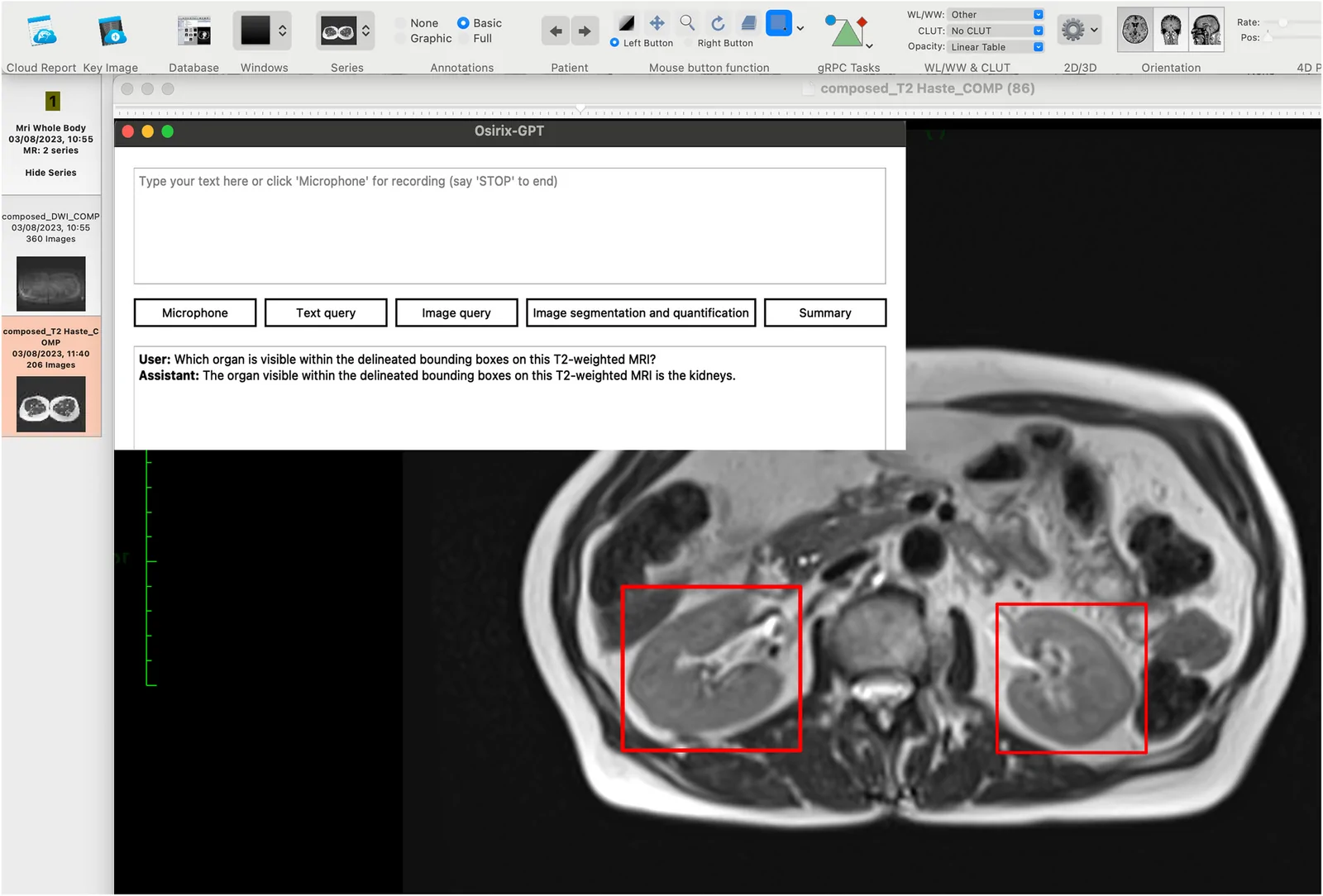
Screenshot of a T2w HASTE MRI with ROIs (red bounding boxes) outlining both kidneys, displayed in the OsiriX 2D DICOM viewer alongside AI-generated text in the OsiriXGPT GUI. In this example, the query combines textual and visual information. The text prompt “*Which organ is visible within the delineated bounding boxes in this T2-weighted MRI?*” was processed along with the T2w image and ROIs using the “Image query” control in the *user control* panel. The input text, images, and ROIs were transferred from OsiriX to the Python environment via the OsiriXgrpc API plug-in, where the OpenAI API is installed to enable inference by OpenAI’s VLMs on cloud-based servers. The OsiriXGPT interface processed the query successfully, and the VLMs provided the correct answer

**Test 2**: The model accurately identified both kidneys within the provided bounding boxes, and the response was generated within 3 s (Fig. 4), confirming the successful integration of the OpenAI API for VLMs with data queried from the OsiriX 2D viewer and the text prompt entered by the user in the GUI’s *input dialogue*. Here, rather than focusing on the algorithm’s overall accuracy, we provide insight into whether our tool could be used by medical imaging professionals, including research radiographers, to generate text from images.

**Fig. 5:**
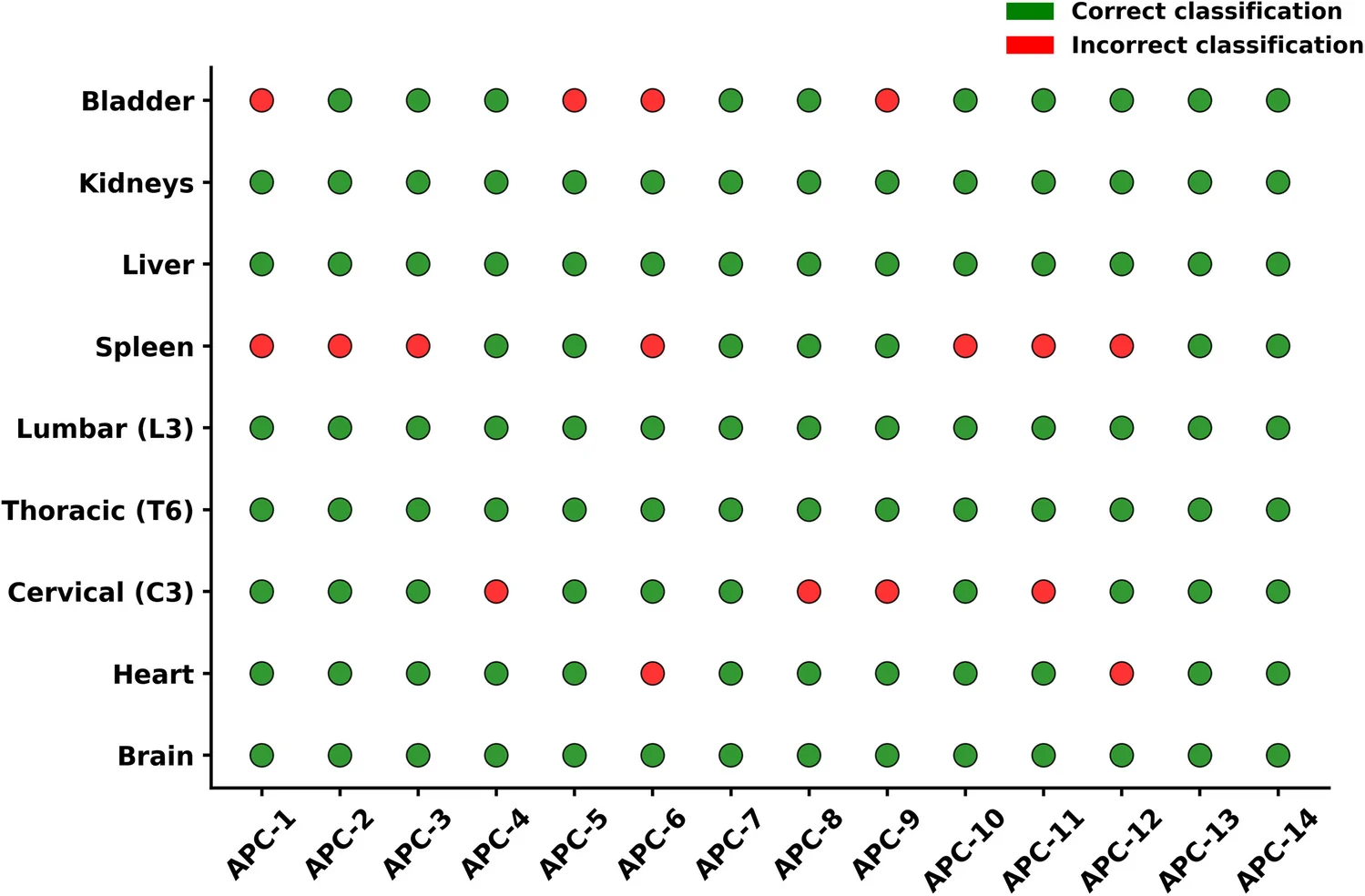
Scatter plot showing the classification performance of OpenAI’s VLMs in identifying multiple anatomical structures using user-defined text prompts, T2w HASTE images, and ROIs outlining the anatomical structures in patients diagnosed with Advanced Prostate Cancer (APC). Each point represents the classification outcome for a specific patient-anatomical structure pair. The model demonstrated promising performance, achieving an overall classification accuracy of 86.5% (109 correct classifications out of 126 attempts)

We observed variability in the performance of VLMs when classifying anatomical structures such as the bladder, kidneys, liver, spleen, vertebral bodies (L3, T6, C3), heart, and brain on T2-w HASTE images in patients diagnosed with APC using OsiriXGPT (see Fig. 5). While the model performed well in classifying most structures across all patients, its accuracy was lower for the heart, cervical spine, bladder, and spleen. The heart was correctly classified in 85.7% of cases (12/14), the cervical spine (C3) in 71.4% (10/14), the bladder in 71.4% (10/14), and the spleen in only 50% (7/14).

Misclassification patterns included the cervical C3 vertebra being identified as the spinal cord without recognising its bony structure, the spleen frequently being mistaken for the kidney, and the bladder occasionally misclassified as the prostate gland.

At the patient level, the model showed its lowest performance in one patient, where three structures were misclassified, while for four patients, all structures were correctly classified. Across all structures and patients, the model achieved an overall average classification accuracy of 86.5% (109/126).

**Fig. 6:**
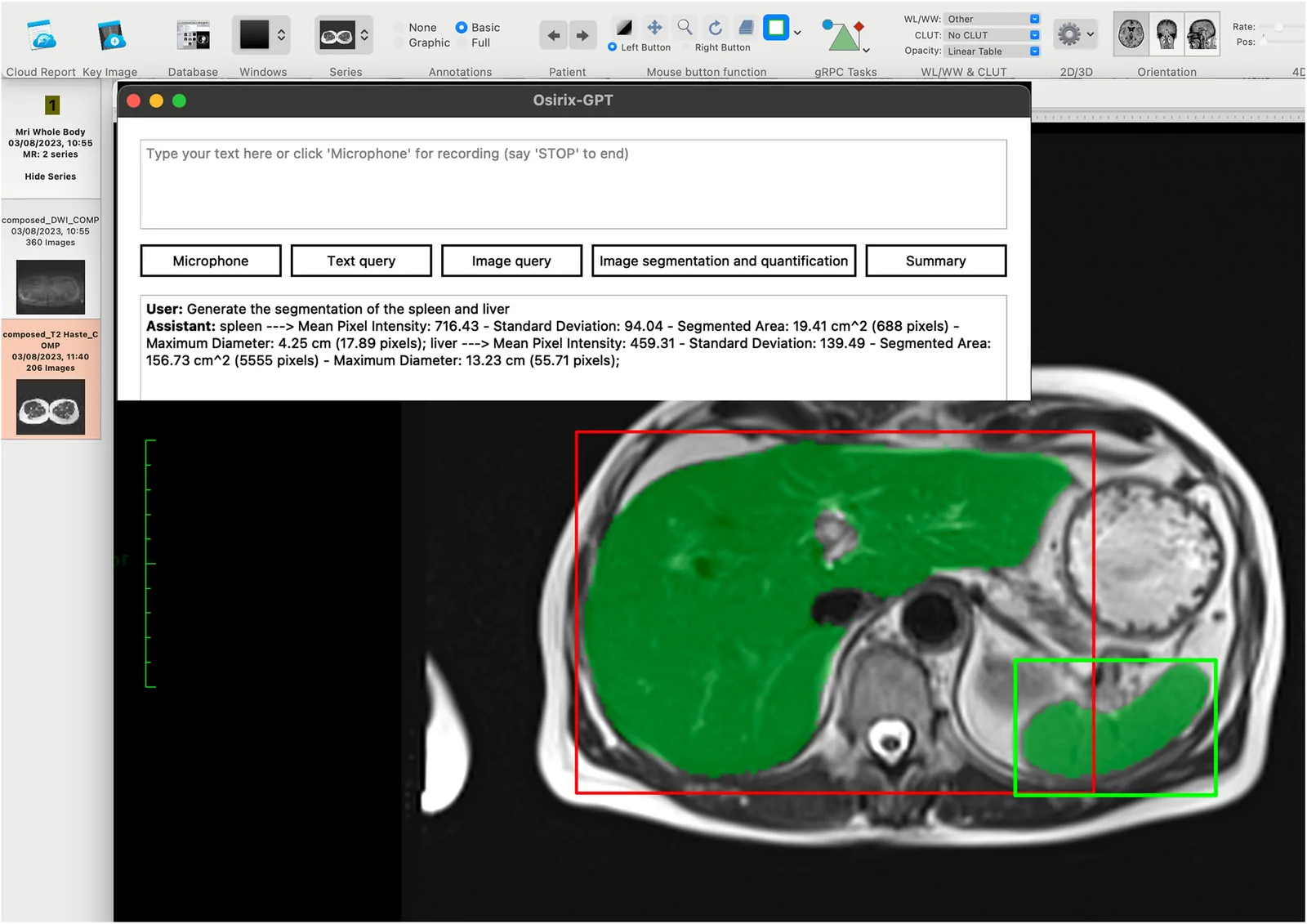
Screenshot of a T2w HASTE MRI with AI-generated delineations of the spleen and liver displayed in the OsiriX 2D DICOM viewer. The delineations were generated using the Segment Anything Model (SAM), which processes images based on ROI prompts. In this example, bounding boxes for the spleen (green) and liver (red) were easily defined using OsiriX ROI tools. The text prompt “*Generate the segmentation of the spleen and liver*” was entered into the OsiriXGPT GUI *input dialogue*, and the process was initiated using the “Image segmentation and quantification” control. The input text, images, and associated ROIs were transferred from OsiriX to the Python environment, where the segmentation model is installed, via the OsiriXgrpc API plug-in. Following model inference, the delineations were displayed in the OsiriX 2D viewer, along with associated measurements (e.g., area and diameter) in the OsiriXGPT GUI *output window*

**Test 3**: OsiriXGPT successfully displayed reliable ROIs on the OsiriX 2D viewer window within 4 s of initiating the segmentation request (Fig. 6). Quantitative measurements, including area, maximum diameter, and signal intensity, were provided in the GUI’s *output window* from delineated regions. This confirmed the successful integration of the SAM model with the OsiriXGPT interface and the accurate visualisation of the segmented masks within the OsiriX DICOM viewer.

**Fig. 7:**
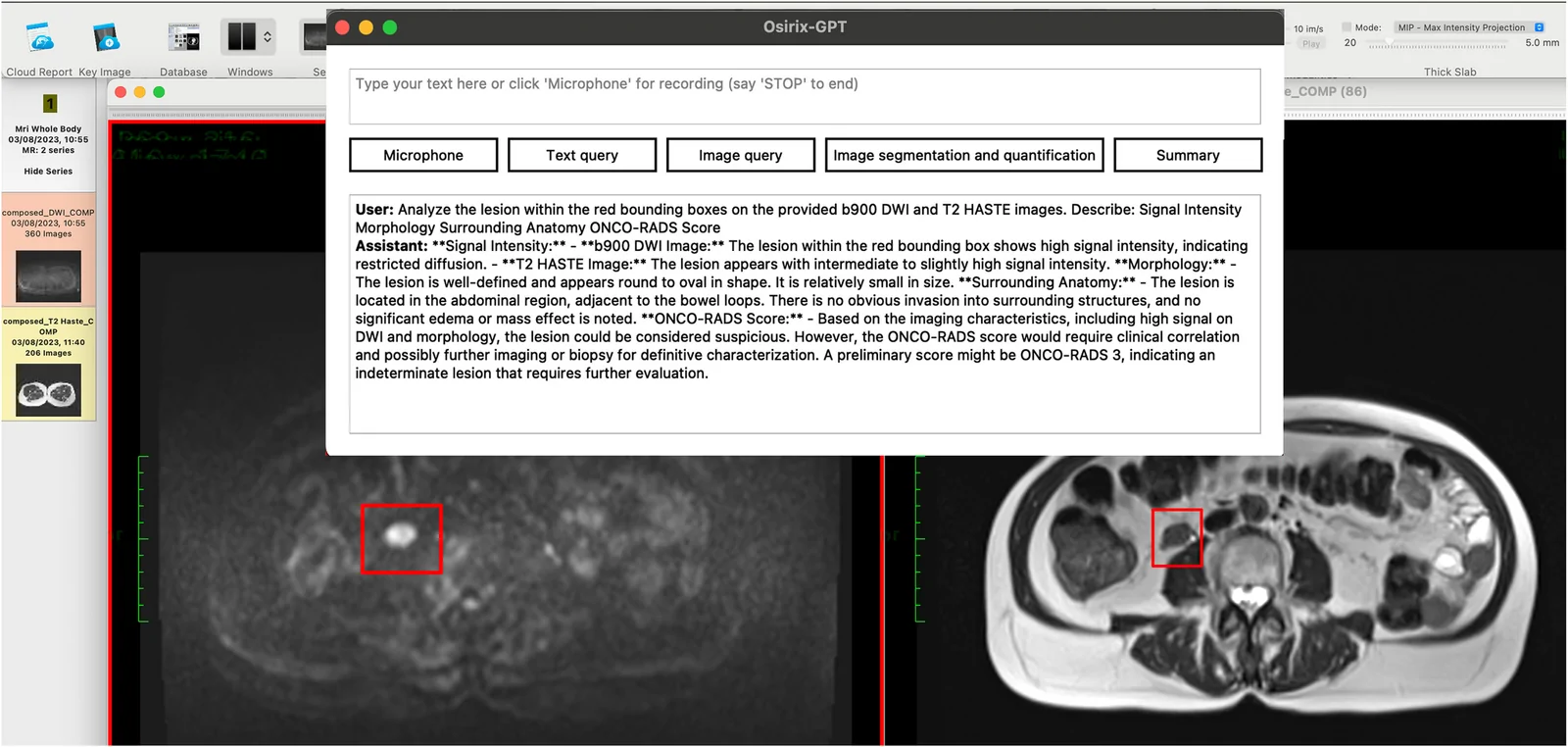
Screenshot of a b900 DWI and T2w HASTE MRI showing a bounding box delineating a suspected retroperitoneal lesion, displayed in the OsiriX 2D DICOM viewer alongside AI-generated text in the OsiriXGPT GUI. Similar to Fig. 4, the query combines textual and visual information. In this example, the visual input included multiple MRI sequences and a bounding box highlighting the lesion to describe its voxel characteristics. The text prompt “*Analyse the lesion within the red bounding boxes on the provided b900 DWI and T2w HASTE images. Describe: signal intensity, morphology, surrounding anatomy, ONCO-RADS score*” was entered into the OsiriXGPT GUI *input dialogue* and processed using the “Image query” control. The OsiriXGPT interface processed the query successfully, and the VLMs generated a response consistent with the clinical findings visible in the MRI sequences

**Test 4**: The AI-generated output correctly identified increased cellularity in the lesion, suggesting potential malignancy due to an abnormal signal in the b900 image (Fig. 7). The surrounding tissue was correctly described by the VLMs as normal, and the final ONCO-RADS score assigned was 3. These findings were confirmed by an expert radiologist in WB-MRI, initially blinded to the results of the ChatGPT inference.

**Fig. 8:**
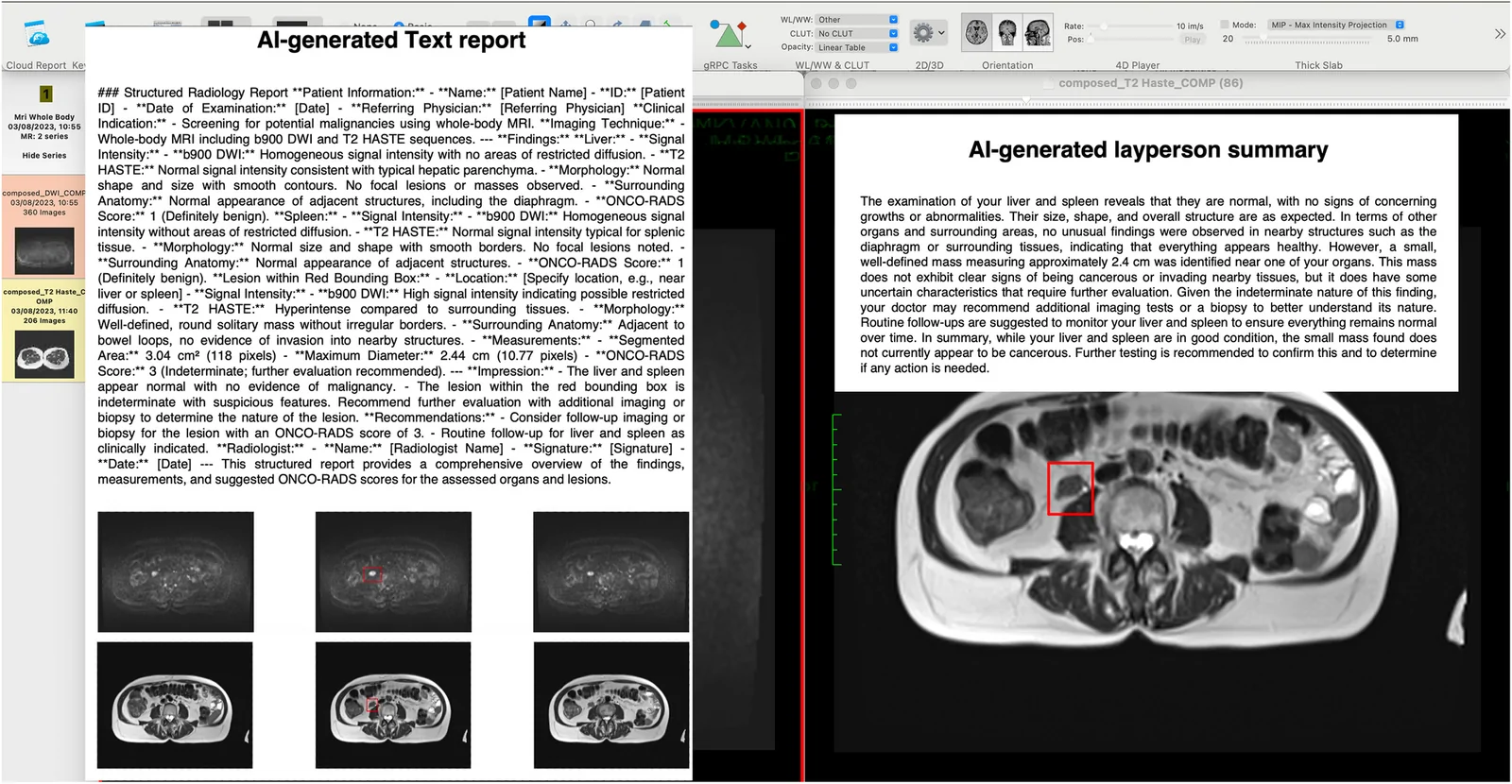
Screenshot of two AI-generated PDF files using OsiriXGPT. The first PDF shows a text-based report recording the request-to-answer dialogue between User and AI Assistant. The second PDF presents a translation for layperson of the same report. To generate the initial report, the user entered the prompt “*AI-generated Text Report*” into the OsiriXGPT GUI *input dialogue* and initiated the process by selecting the “Summary” option from the *control panel*. This PDF also includes b900 DWI and T2w HASTE MRI axial images with superimposed delineations of a suspected retroperitoneal lesion, as displayed in the OsiriX 2D DICOM viewer during the user’s interaction with the GenAI models via the OsiriXgrpc AI plug-in. To generate the layperson summary PDF, the user entered the text prompt “*Translate this AI-generated text-based report with radiological findings into a layperson summary report”* and executed the process again using the “Summary” button

**Test 5**: The dialogue from the prior radiologist-ChatGPT interactions was compiled into an automatically generated report summarising the relevant findings, including the suspected retroperitoneal lesion and the corresponding ONCO-RADS score (Fig. 8). The AI-generated summary (that potentially could be exported as a DICOM structured report and delivered back into the PACS workflow) included the following:

- **Liver**: The liver was of normal size with smooth contours, and no focal lesions were observed. The assigned ONCO-RADS score was 1, indicating a benign finding.
- **Spleen**: The spleen demonstrated normal signal intensity on both b900 DWI and T2-weighted HASTE MRI sequences, with no abnormalities or focal lesions. It was also of normal size and shape, receiving an ONCO-RADS score of 1, signifying a benign result.
- **Lesion**: In contrast, the lesion showed high signal intensity on b900 DWI, indicative of restricted diffusion, and a hyperintense signal on T2-weighted HASTE imaging compared to surrounding tissues. Morphologically, the lesion appeared well-defined and round. The delineated area of the lesion measured 3.04 cm^2^, with a maximum diameter of 2.44 cm. Based on these findings, the lesion was assigned an ONCO-RADS score of 3, warranting further evaluation through additional diagnostic tests.

### OsiriXgrpc Plug-In for WB-DWI SAM

**Fig. 9:**
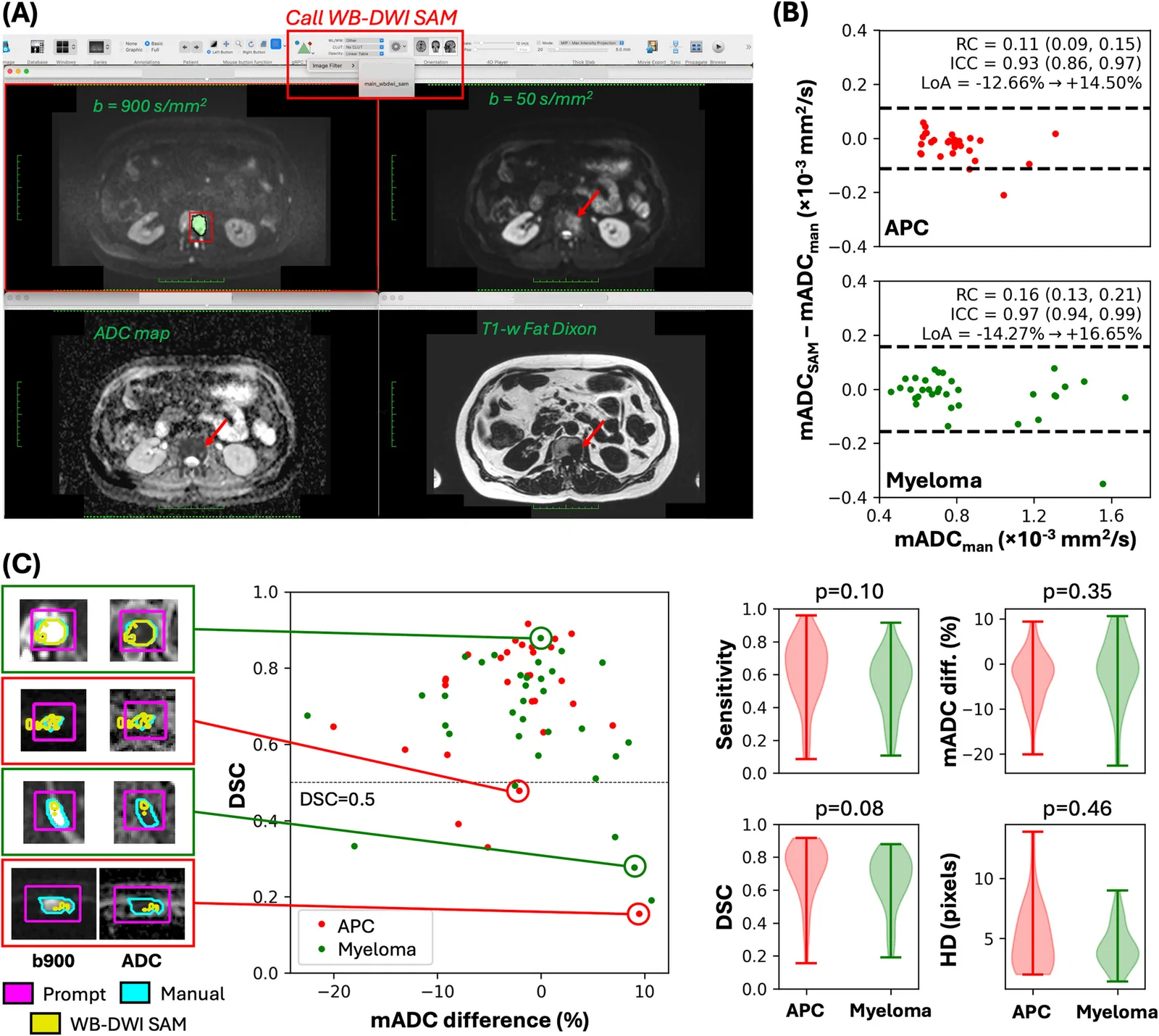
A Example segmentation of a metastatic bone lesion in advanced prostate cancer (APC) using WB-DWI SAM via the OsiriXgrpc API plug-in. The radiologist confirmed disease characteristics across multiple sequences before defining a bounding box (red) on the b = 900 s/mm^2^ image; the resulting segmentation mask was displayed in the DICOM viewer (green mask). (B) Bland–Altman plots comparing median ADC (mADC) estimates from manual single-slice delineations (mADC_man_) and WB-DWI SAM (mADC_SAM_). Results are shown for APC (red) and multiple myeloma (blue), with repeatability coefficients (RC), intra-class correlation coefficients (ICC), and limits of agreement (LoA) shown (95% confidence intervals in parentheses). (C) Left: Examples of tumour delineations across a range of dice-similarity coefficients (DSC), with scatter plots showing the relationship between DSC and percentage mADC difference. Right: violin plots of segmentation losses (e.g., Hausdorff distance, HD), with no significant differences between APC and myeloma in any of these (*p* < 0.05)

A screenshot of our plug-in is presented in Fig. 9A. In total, 30 lesions were assessed for APC (6 patients) and 33 lesions for myeloma (6 patients). Bland–Altman plots, presenting the difference between median ADC (mADC) values from ROIs derived manually and using the WB-DWI SAM model are presented in Fig. 9B in addition to key statistics. In both APC and myeloma, good agreement in mADC is observed with high ICC > 0.9 and LoA at approximately − 15% to 17%. Furthermore, the CoV was calculated to be 4.05% for APC and 5.67% for myeloma, within the values typically observed in test–retest baseline studies of ADC measured in previous studies of extracranial soft-tissue tumours (CoV range from 1.7% to 6.3% across nine independent cancer studies [44]). Distributions of morphological metrics comparing binary segmentations are compared in Fig. 9C. 54/63 lesions demonstrated good DSC > 0.5, and the following median metrics (95% interval in parentheses:

- **APC**: DSC = 0.77 (0.36–0.88), sensitivity = 0.65 (0.22–0.85), HD = 5.00 (2.11–9.66).
- **Myeloma**: DSC = 0.68 (0.31–0.84), sensitivity = 0.59 (0.22–0.77), HD = 4.00 pixels (1.91–7.37).

No significant differences between distributions of morphological metrics for APC and myeloma were found.

## Discussion

One of the key technical advantages of OsiriXgrpc is its versatility, allowing developers to implement client messages in any programming language supported by gRPC with minimal additional effort. At the time of writing, gRPC supports a wide range of languages, including C#, C +  +, Dart, Go, Java, Kotlin, Node.js, Objective-C, PHP, Python, and Ruby. Moreover, gRPC enables the secure transfer of messages over a network through SSL encryption, which is particularly crucial when the OsiriXgrpc client operates over a network rather than on the same machine as the OsiriX server. This flexibility empowers developers and the open-source community to efficiently introduce new features to OsiriX and its open-source and free derivative software, Horos (available from https://horosproject.org/).

The potential benefits of OsiriXgrpc for agile development and deployment of AI-driven models are substantial. These benefits include leveraging domain knowledge from clinicians within a familiar user interface to build better predictive models, enabling faster experimental iterations, and facilitating the seamless integration of AI technologies into clinical research workflows. An initial learning curve to using this technology is the implementation of Google’s protobuf language. Whilst this provides the most flexibility, we have also implemented a sister repository, pyOsiriX (available from [*blinded for review*] as a major update to our prior implementation. This library provides a much more Pythonic interface to OsiriXgrpc and is thus our suggested route for those seeking to rapidly develop AI-enabled plugins moving forward.

Beyond research applications, OsiriXgrpc could help integrate AI technologies into clinical practice. Since OsiriX is already CE-marked and FDA-approved, certifying OsiriXgrpc as a platform would reduce the regulatory burden of deploying AI-driven models in healthcare. Instead of requiring approval for both the AI model and its deployment platform, only the model would need certification, streamlining the process and cutting costs for healthcare providers. Additionally, this platform could benefit small private radiology practices in high-income countries that fall below the threshold for installing a PACS from major vendors. It may also support healthcare systems in LMICs, where OsiriX is already used for clinical reporting. Approximately 70% of global cancer deaths occur in LMICs [45]. There is an urgent need to increase access to state-of-the-art resources within LMICs that can improve early diagnosis rates [46], and account for the paucity of specialists in rare diseases including sarcoma [47]. Leveraging technology for “image analysis and pattern recognition for pathology and radiology” was recently highlighted as one of the five key research priorities for LMICs over the next decade [48]. By leveraging the OsiriXgrpc API plug-in, OsiriX provides a lightweight, adaptable solution that enhances access to AI-assisted radiology while lowering financial and technical barriers. By making AI-driven imaging more accessible worldwide, OsiriXgrpc has the potential to drive the global adoption of AI-powered radiology.

Through OsiriXgrpc, we have developed and released OsiriXGPT, an innovative interface that integrates OpenAI’s LLMs and VLMs creating a streamlined radiology assistant within OsiriX/Horos. Initial verification tests have demonstrated the toolkit’s capabilities, including: (i) providing radiologists with a text- or voice-based interface to state-of-the-art OpenAI LLMs for accessing information about disease-specific reporting standards; (ii) leveraging VLMs to enhance the interpretation of outlined regions in images within OsiriX/Horos, thereby assisting non-experts in image analysis; (iii) enabling semi-supervised delineation of image structures and the extraction of image statistics through foundation models, including SAM and a custom fine-tuned version for WB-DWI scans; and (iv) offering insights into regions of potential concern within whole-body MRI studies.

There is growing interest in the role of WB-MRI for cancer screening in both healthy individuals and high-risk populations. However, while WB-MRI remains a highly specialized field, other widely available radiological scans can now generate datasets that are growing in size and complexity, which makes interpretation and reporting more challenging for radiologists. These assessments often require labour-intensive manual measurements, such as those used in RECIST or Choi criteria, or advanced imaging techniques (i.e. radiomics or emerging VLMs) to extract insights that exceed radiologists’ capabilities. Given that modern radiological scans often comprise hundreds of images, significantly increasing the complexity of interpretation and the number of measurements required, and the increasing burnout among radiologists across healthcare institutions, the successful implementation of AI as a diagnostic aid will provide valuable support towards the widespread adoption of these imaging techniques. We hope that OsiriXgrpc and OsiriXGPT will serve as free and valuable platforms for researchers to explore the impact of AI tools on the radiological workflow in this context and beyond. By harmonizing the environment in which these technologies are tested and validated, these platforms could help bridge the gap between AI research and clinical implementation. While initially designed for research PACS environments, the insights gained from these tools could accelerate the integration of foundation multi-modal biomedical AI models into clinical PACS systems, ultimately supporting real-world adoption.

It is important to note that this study did not focus on evaluating the accuracy of AI-generated outputs but rather on optimizing the workflow that facilitates interaction between radiologists and GenAI models through our OsiriXGPT interface. This study has several limitations that should be acknowledged. Firstly, although the tool is designed to improve radiological workflows, we did not perform a head-to-head comparison with conventional reporting systems. Future work should therefore quantify reporting time, number of interaction steps reduced, and cognitive load to demonstrate potential gains in efficiency and accuracy [49]. Secondly, we did not perform a usability study using validated metrics (e.g., System Usability Scale, NASA-TLX) or structured feedback from radiologists to evaluate ease of use, learning curve, and interface clarity [50]. Thirdly, we did not expose model confidence scores or uncertainty estimates to users; integrating such outputs into the interface will be essential to promote trust and safe adoption of AI tools in radiology practice [51]. Fourthly, while we provide initial quantitative results for a custom fine-tuned segmentation model for medical images (WB-DWI SAM), further evaluation of its OsiriXgrpc plug-in with multiple readers, fully volumetric disease segmentation and more diverse clinical scenarios remains future work. Achieving these goals will require active collaboration with the radiological community to establish the most effective methods for displaying and interpreting such results in clinical practice.

Data privacy is a critical concern in this context. Our approach involves implementing a gatekeeper process, connected via gRPC, running on the same machine where the data are stored. This process ensures that any patient-identifiable information is stripped from images before they are uploaded to the OpenAI cloud. However, the OCR-based redaction module achieved 90% accuracy in our tests and so we advocate that users perform an additional manual checks of generated JPEG images from DICOM prior to upload to any cloud-based platform, including OpenAI. It is important to note that the parameters we chose we those suggested by the developer, and further optimization may improve these results in future studies. Beyond image data, it is challenging to eliminate the risk of unintentional disclosure of patient information through prompts entered by radiologists. One potential solution could involve using on-system natural language processing (NLP) to automatically identify and remove any accidentally included patient-identifiable information in the prompts [52].

Alternatively, hosting the AI models locally on the same system could mitigate this risk entirely. However, the latter approach may limit the flexibility and up-to-date functionality of the models used by OsiriXGPT, as locally hosted models may not always reflect the latest advancements or updates available in the cloud-based versions. Local deployment is also limited by hardware and memory requirements: while the gRPC server can connect to GPU-enabled workstations, inference can also run locally on Mac systems using Apple’s Metal Performance Shaders (MPS) backend for GPU acceleration. Moreover, many institutions and users may lack access to such powerful, and often expensive, hardware with sufficient memory, which could limit the scalability of our solution.

In conclusion, we developed an open-source plug-in that integrates GenAI models into a CE-marked and FDA-approved DICOM viewer. Our work is primarily a software integration and workflow tool, providing a gRPC-based interface that enables real-time interaction with foundation models. The system allows users to submit multimodal prompts and receive AI-generated responses directly within the imaging workflow. As a result, our solution provides a research-ready platform for radiologists to evaluate cutting-edge GenAI models in clinical trials and exploratory studies, while also enabling investigation of their potential role in routine clinical practice.

## Supplementary Information

Below is the link to the electronic supplementary material.

Supplementary file1 (DOCX 1.32 MB)

## Data Availability

Data can be shared upon request from the authors. However, this is subject to the establishment of an appropriate data-sharing agreement, given the sensitive nature of patient information involved.

## Abbreviations

**APC**: Advanced Prostate Cancer
**GDPR**: General Data Protection Regulation
**GenAI**: Generative Artificial Intelligence
**Grpc**: Google Remote Procedure Call
**GUI**: Graphical User Interface
**HASTE**: Half-Fourier Acquisition Single-Shot Turbo Spin Echo
**LLMs**: Large-Language Models
**LMICs**: Low/middle income countries
**NLP**: Natural Language Processing
**PACS**: Picture Archiving and Communication System
**RIS**: Radiology Information System
**ROIs**: Regions of Interest
**SAM**: Segment Anything Model
**T2w**: T2-weighted
**VLMs**: Vision-Language Models
**WB-DWI**: Whole-body diffusion-weighted MRI
**WB-MRI**: Whole-body MRI

## Declarations

### Note to Editor (regarding anonymised content):

The manuscript refers to an anonymised GitHub repository (“[*blinded for review*]”) that is essential for understanding and evaluating the core contributions of this work. The actual repository is available here: https://github.com/osirixgrpc/osirixgrpc

The related prior tool, pyOsiriX, mentioned in the manuscript as a previous solution, is also hosted at: https://github.com/osirixgrpc/pyosirix

These links are redacted in the manuscript text to comply with double-blind review policy and can be reinstated upon acceptance.
