# Supplementary material for: OsiriXGPT: An Innovative AI Co-pilot Plug-In for Seamless Deployment of Generative AI Models in Scan-to-Scan Reporting Workflows
Source: J Imaging Inform Med. 2025 Oct 16;39(5):3881–97. doi: 10.1007/s10278-025-01712-2 (PMC13694589; doi:10.1007/s10278-025-01712-2)
Supplement: Supplementary file 1 [file 10278_2025_1712_MOESM1_ESM.docx]

**Supplementary Information**

**S1. Fine-tuning a foundation model to delineate cancer lesions from Whole-body Diffusion Weighted MRI (WB-DWI)**

**S1.1 Methods**

We fine-tuned the Segment Anything Model (SAM) [37] on a single-centre anonymised retrospective WB-DWI cohort consisting of 144 scans from 72 patients diagnosed with advanced prostate cancer (APC), each of whom underwent both baseline and post-treatment scans. The cohort was split into training (58 patients, 116 scans) and validation (14 patients, 28 scans) datasets. To evaluate the fine-tuned model (hereafter referred to as WB-DWI SAM), we tested it on three independent retrospective and anonymised WB-DWI cohorts: (i) 10 patients with confirmed APC, (ii) 16 patients with multiple myeloma (MM), and (iii) 13 patients with lymphoma. Experienced radiologists manually delineated cancer lesions across the body in all training, validation, and test sets, providing the reference standard for evaluation.

**S1.2 Model development**

SAM consists of three main components: an image encoder, a flexible prompt encoder, and a fast mask decoder based on Transformer vision models. We froze the image and prompt encoder, training only the mask encoder's weights [19]. Data pre-processing involved generating 2D axial RGB images from WB-DWI datasets, with three channels: (i) S0 image and (ii) ADC map, both calculated from at least two b-values (typically b=50 and b=900 s/mm²) [53]; and (iii) zeros in the final channel. Images and masks were resampled to a matrix size of 256x256 with a resolution of 1.6x1.6mm. Image embeddings were extracted from the 2D axial RGB images using the SAM encoder, generating a 256x64x64 tensor. Bounding box prompts were created by introducing random perturbations of +/- 15 pixels to each side. For model fine-tuning, we used Adam optimization with a learning rate of 10^-5^. Input data included RGB images, image embeddings, and bounding boxes. The model was trained by minimizing the dice loss function over 300 epochs, with a batch size of 1, and the network’s final layer employed a sigmoid function. All algorithms were implemented in Python (v3.9.19) using PyTorch v2.3.0, running upon a Windows platform (v.10.0.19) accelerated by an NVIDIA RTX6000 GPU (Santa Clara, California, US)

**S1.3 Results**

WB-DWI SAM outperformed the baseline SAM in delineating cancer lesions from WB-DWI test scans. The fine-tuned model achieved an average patient-wise dice score of 0.68 and precision of 0.69, compared with 0.47 and 0.32, respectively, for the baseline SAM.

**S1.4 OsiriXgrpc plug-in for WB-DWI SAM**

We developed and deployed an OsiriXgrpc plug-in that integrates the fine-tuned WB-DWI SAM model directly into the OsiriX viewer. Users can access the plug-in when reviewing multi-parametric whole-body MRI study including DWI (b=50-900 s/mm² and ADC map) and T1-weighted or T2-weighted sequences [54]. To generate a segmentation mask, the user draws a bounding box around the lesion of interest (typically on b=900 s/mm²), after which the WB-DWI SAM plug-in is called for inference. As the first step, b-value images are sent from the OsiriX 2D viewer to the Python client for pre-processing as described in Section S1.2.

To support 3D lesion segmentation, the plug-in allows multiple bounding boxes to be placed at different slice levels (e.g., start, middle, and end of the lesion). These bounding boxes are interpolated across the volume, and inference is performed slice-by-slice in a single call. The resulting masks are automatically displayed in the OsiriX viewer, where users can review and edit them if needed. This approach enables volumetric lesion measurements while reducing the number of manual interactions otherwise required for slice-by-slice bounding box prompting and inference.

**Supplementary Figures**

Figure S1. Example DICOM images showing burned-in PHI (patient ID and date of birth, DOB) before and after applying the OCR-based redaction algorithm. One failed test is also shown (red arrow).
